# Surface and Thermal Characterization of Cotton Fibers of Phenotypes Differing in Fiber Length

**DOI:** 10.3390/polym13070994

**Published:** 2021-03-24

**Authors:** Zhongqi He, Sunghyun Nam, David D. Fang, Huai N. Cheng, Jibao He

**Affiliations:** 1 USDA-ARS, Southern Regional Research Center, 1100 Robert E Lee Blvd., New Orleans, LA 70124, USA; sunghyun.nam@usda.gov (S.N.); david.fang@usda.gov (D.D.F.); hn.cheng@usda.gov (H.N.C.); 2 Coordinated Instrument Facility, Tulane University, New Orleans, LA 70118, USA; jhe1@tulane.edu

**Keywords:** cotton, Fourier transform infrared spectroscopy, ligon-lintless mutation, micromorphology, short fiber mutant, surface composition

## Abstract

Cotton is one of the most important and widely grown crops in the world. Understanding the synthesis mechanism of cotton fiber elongation can provide valuable tools to the cotton industry for improving cotton fiber yield and quality at the molecular level. In this work, the surface and thermal characteristics of cotton fiber samples collected from a wild type (WT) and three mutant lines (*Li*_1_, *Li*_2_-short, *Li*_2_-long, *Li*_2_-mix, and *li_y_*) were comparatively investigated. Microimaging revealed a general similarity trend of WT ≥ *Li*_2_-long ≈ *Li*_2_-mix > *Li*_1_ > *Li*_2_ short ≈ *li_y_* with Ca detected on the surface of the last two. Attenuated total reflectance Fourier transform infrared (ATR FT-IR) spectroscopy and thermogravimetric measurements also showed that *Li*_2_-short and *li_y_* were more similar to each other, and *Li*_2_-long and *Li*_2_-mix closer to WT while *Li*_1_ was quite independent. FT-IR results further demonstrated that wax and amorphous cellulose were co-present in fiber structures during the fiber formation processes. The correlation analysis found that the FT-IR-based maturity parameter was well correlated (*p* ≤ 0.05) to the onset decomposition temperature and all three weight-loss parameters at onset, peak, and end decomposition stages, suggesting that the maturity degree is a better parameter than crystallinity index (CI) and other FT-IR parameters that reflect the thermal stability of the cotton fiber. In summary, this work demonstrated that genetic mutation altered the surface and thermal characteristics in the same way for *Li*_2_-short and *li_y_*, but with different mechanisms for the other three mutant cotton fiber samples.

## 1. Introduction

Cotton is one of the most important and widely grown crops in the world [1]. It is a well-traded agricultural commodity primarily used for textile fiber purposes. Cotton fibers are unicellular seed trichomes that differentiate from the epidermal cells of developing cotton ovules. They are produced from the protodermal cells on the outer integument layer of a fertilized cottonseed, taking approximately 1.5~2 months to become fully mature or developed fibers depending on environmental conditions. The fiber development process roughly occurs with some overlaps in a series of initiation, elongation, secondary cell wall biosynthesis, and maturation [2]. Cotton fiber length is largely determined at the elongation stage. As fiber length is very important to the quality of textiles, understanding the genetics and physiology of cotton fiber elongation can provide valuable tools to the cotton industry by targeting genes or cropping factors responsible for fiber elongation [3,4]. Cotton fiber mutants provide such a resource for the elucidation of fiber development mechanisms owing to the structural, morphological, and biochemical variances in the relevant fiber cells [5,6].

**Figure 1:**
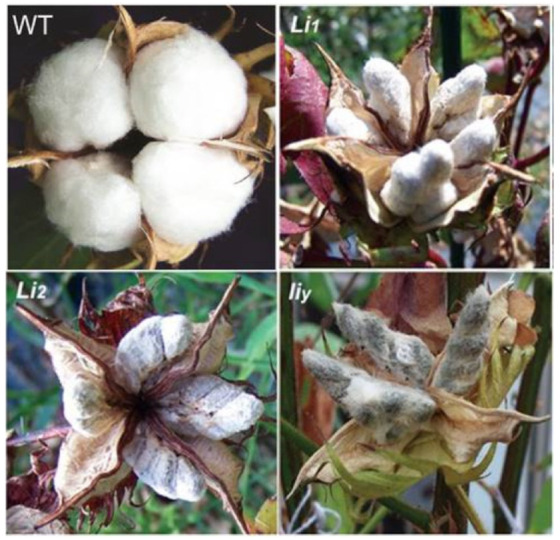
Fiber phenotypes of wild-type and three mutant lines.

**Figure 2:**
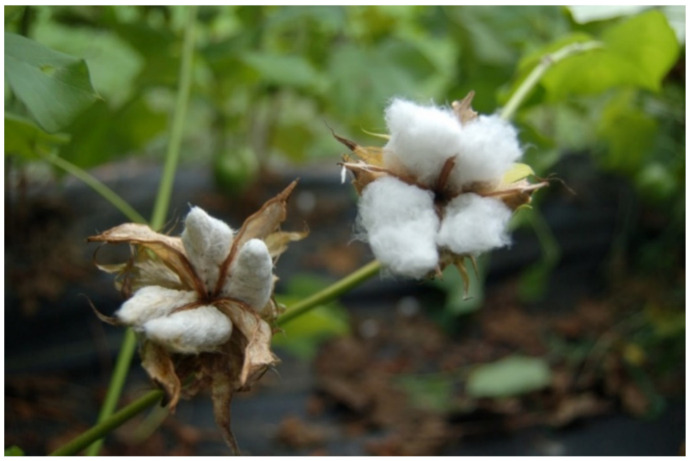
A *Li*_2_ cotton mutant plant with mixed fiber phenotypes. Left boll, *Li*_2_-*mix* (i.e., short fiber in a *Li*_2_ plant with mixed phenotypes); right boll, *Li*_2_-*long*.

There are numerous naturally occurred and human-made fiber mutants that display various fiber phenotypes [7]. These include, among others, fiberless mutant lines MD17, SL1-7-1, and XZ142w [8,9]; seeds with only lint fibers and no fuzz fibers in the naked seed lines *N_1_* [10] and *n_2_* [11]; and seeds that are described as extremely short lint fibers in the Ligon lintless-1 (*Li*_1_) [12], Ligon lintless-2 (*Li*_2_) [13], *Li_x_* [14] and *li_y_* [4] mutant lines. Among the four mutant lines, we selected three mutants, (i.e., *Li*_1_, *Li*_2_, and *li_y_*) in this study. All three mutants had seed fibers that were extremely short (<6 mm) compared to the wild-type (WT) fibers that are typically greater than 20 mm in length (Figure 1). Both *Li*_1_ and *Li*_2_ mutations are controlled by a single dominant gene while *li_y_* mutation is controlled by one single recessive locus. Although both *Li*_1_ and *Li*_2_ are monogenic mutants which exhibit an early cessation of fiber elongation [3,5,15], *Li*_1_ mutant exhibits pleiotropy in the form of severely stunted and deformed plants in both the homozygous dominant and heterozygous state, while the *Li*_2_ mutant plants appear healthy and morphologically identical to the homozygous recessive wild-type plants with the exception of shorter seed fibers [16]. In addition, a mixed phenotype (i.e., cotton bolls with short fiber and normal length fiber present in the same plant) (Figure 2) resulting from incomplete dominance has been observed in a *Li*_2_ mutant but not in *Li*_1_ [17]. The *Li*_1_ mutation is caused by a Gly65Val substitution in an actin gene on chromosome D04, which disrupts cell polarity and F-actin organization resulting in dwarf, lintless cotton plants [15]. The *Li*_2_ mutation is located within a terminal deletion of chromosome 18 (also called chromosome D13) [18]. A comparative study shows that at least seven of the 10 putative fiber development genes in the deletion region showed a higher expression in the wild type than in a *Li*_2_ mutant during the fiber development stages. The *li_y_* mutant was created by treating the seeds of the cotton line MD15 with ethyl methanesulfonate [4,19]. A *li_y_* plant is short and stunted. The *li_y_* gene locus is not allelic to either *Li*_1_ or *Li*_2_ [6]. A recent study indicates that a tetratricopeptide repeat-like superfamily protein gene (Ghir_A12G008870) on chromosome A12 is responsible for the *li_y_* short fiber phenotype [4].

Among the many genomic studies, there are limited reports which also measured selective fiber structural and quality parameters. Gilbert et al. [3] conducted microscopic image analysis, found that *Li*_1_ fibers at 28 days post anthesis (DPA) were in general “thicker” than WT fibers due to the early cessation of fiber elongation. Hinchliffe et al. [20] obtained the scanning electronic microscopic (SEM) images of developing *Li*_2_ mutant and WT fibers and ovules but found no evident differences between the WT and mutant fibers. Naoumkina et al. [19] reported that the parameters of fiber mean length, short fiber content (SFC), fineness, and maturity ratio of *li_y_* fiber significantly inferior to WT fiber (*p* < 0.05). However, no statistically significant (*p* > 0.05) difference in the parameter of micronaire is observed between the two cotton fibers. Those morphological characterizations helped the genomic studies shed novel light on cotton fiber elongation, suggesting multiple genes coordinately regulated processes involving cell wall development and cell elongation [18].

In this work, we collected the matured fiber samples from a WT cotton and three cotton mutants producing short lints (*Li*_1_, *Li*_2_, and *li_y_*). For the *Li*_2_ mutant, we separated the fibers into three types: *Li*_2_-short, *Li*_2_-long, and *Li*_2_-mix. We conducted the surface characterization of these fiber samples by advanced microscopic technologies and attenuated the total reflection Fourier transform infrared (ATR FT-IR) spectroscopy. The purpose of this work was to systematically compare the surface and thermogravimetric characteristics of these samples for a better understanding of differences in fiber quality between these samples. The eventual goal was to increase our knowledge of gene expression and physiology of cotton fiber, thus finding those fiber-related trait loci with functionally diverse but coordinately regulated genes for cotton elongation and quality.

## 2. Materials and Methods

### 2.1. Materials and Sample Preparation

*Li*_1_, *Li*_2_, and *li_y_* plants were planted in a field in New Orleans, Louisiana, in 2019. Each mutant line was planted in a plot of 1 m (width) × 18 m (length). The soil type is Aquent dredged over alluvium in an elevated location to provide adequate drainage. The space between plants was 30 cm. Common farm practice was applied during the growing season. Cotton bolls in *Li*_1_ and *li_y_* mutant plants were quite homogenous, and all demonstrated a short fiber phenotype. We collected fibers from bolls in the middle part of a plant. For *Li*_2_ mutant plants, we observed two types of plants. The first type had short fiber bolls in the entire plant. We collected cotton bolls from this type of plant, and designated its fiber as *Li*_2_-short. The second type plant had cotton bolls with a short fiber and normal fiber length in the same plant (Figure 2). We separately collected bolls, and designated *Li*_2_-mix as the short fibers from type 2 plants, and *Li*_2_-long as the normal length fiber collected from the same plant. About 30 bolls from 10 plants of each sample were randomly collected from fields and stored into bags for further treatments and testing. The lints of 10–30 bolls were manually separated from seeds in the laboratory, and stored at room temperature (around 22 °C) until use. The replicate numbers of lab testing were listed with each specific measurement.

### 2.2. Microscopically Morphologic Measurements

For optical microscopic images, cotton fibers were carefully placed on a sample slide. The slide was subjected to polarized light, and images were taken using a digital microscope (KH-8700, Hirox, Hackensack, NJ, USA).

For SEM images, an 8 mm × 12 mm double-side sticky carbon tape was placed on an aluminum stud. A thin layer of cotton fibers was gently attached to the tape. The cotton sample was coated with about 3 nm thickness of carbon using Cressington 208HR Sputter Coater. The cotton samples were observed and imaged with Hitachi S-4800 Field Emission Scanning Electron Microscope, operating at 3 kV.

SEM-energy dispersive spectroscopy (EDS) experiments were performed with Hitachi S-3400 SEM and Oxford INCA EDS System at 20 kV. All EDS spectrums were collected with INCA Energy mode at 100x magnifications. For EDS quantitative data, CaCO_3_, SiO_2_, MAD-10 Feldspar, and Wollastonite were used as the calibrating standards of elements C, O, K, and Ca, respectively. The metal concentrations were derived using an iterative procedure. This iterative routine continued until the successive estimates in weight % differed by <0.1% (the methodical/systematic error value).

### 2.3. Water Contact Angle Measurement

The contact angle of a water drop on fiber webs was measured using contact angle analysis equipment (VCA Optima XE, AST Products, Inc., Billerica, MA, USA). A 0.5 μL drop of distilled water was syringed onto the web. The image of the drop was immediately captured and analyzed to yield a contact angle. The contact angles on five different areas were measured and their average value was presented.

### 2.4. Attenuated Total Reflection Reflectance Fourier Transform Infrared (ATR FT-IR) Spectroscopy

ATR FT-IR spectra were measured using a Vertex 70v FTIR spectrometer (Bruker Daltonics, Billerica, MA, USA) equipped with a MIRacle ATR accessory (Pike Technologies) that incorporated a diamond crystal plate as the reflector. Five measurements at different locations for each sample were collected over the spectrum range of 4000–600 cm^−1^ at 4 cm^−1^ resolution and with 16 scans. All spectra were presented in absorbance. Based on the following FTIR three-band ratio algorithms [21,22], the crystallinity index (*CI*_IR_) and maturity (*M*_IR_) were calculated as

*M*_IR_ (%) = (*R*_1_ − *R*_1,S_)/(*R*_1,L_ − *R*_1,S_) × 100 (1)

where *R*_1_ represents the intensity ratio, that is, (*I*_956_ − *I*_1500_)/(*I*_1032_ − *I*_1500_), and *R*_1,S_ is the smallest *R*_1_ (=0.14), and *R*_1,L_ is the largest *R*_1_ (=0.59).

*CI*_IR_ (%) = (*R*_2_ − *R*_2,S_)/(*R*_2,L_ − *R*_2,S_) × 100 (2)

where *R*_2_ represents the intensity ratio, that is, (*I*_708_ − *I*_800_)/(*I*_730_ − *I*_800_), and *R*_2,S_ is the smallest *R*_2_ (=0.14), and *R*_2,L_ is the largest *R*_2_ (=3.40). The correlation of *CI*_IR_ with CI obtained from X-ray diffraction measurement has been established [21,22].

### 2.5. Thermogravimetric (TG) and Differential Thermogravimetric (DTG) Measurements

Thermogravimetric (TG) and differential thermogravimetric (DTG) analyses were carried out using a TGA Q500 thermal gravimetric analyzer (TA Instruments, New Castle, DE, USA) under a nitrogen atmosphere. The nitrogen flow into the furnace was maintained at a rate of 90 mL/min. Approximately 7 mg of the sample placed in a platinum pan were heated from 30 ± 5 °C to 1000 °C with a heating rate of 10 °C/min. TG and DTG thermograms were analyzed using Universal Analysis 2000 software (TA Instruments). Three measurements were performed. Char yield was determined by weighing the sample before and after pyrolysis. Three measurements were performed.

### 2.6. Data Treatments and Statistical Analysis

Data are presented in the format of average ± standard deviation. Average values, if significantly different (*p* ≤ 0.05) based on the Wilcoxon rank-sum tests, are indicated by different letters on the plots and tables. Principal component analysis (PCA) calculations were performed for FT-IR spectra using OriginLab software (Northampton, MA, USA). The correlation coefficients analysis between the two sets of ATR FT-IR and thermal parameters was performed using the data analysis package in Microsoft Excel 2016.

## 3. Results and Discussion

### 3.1. Optical Microscopic Images

**Figure 3:**
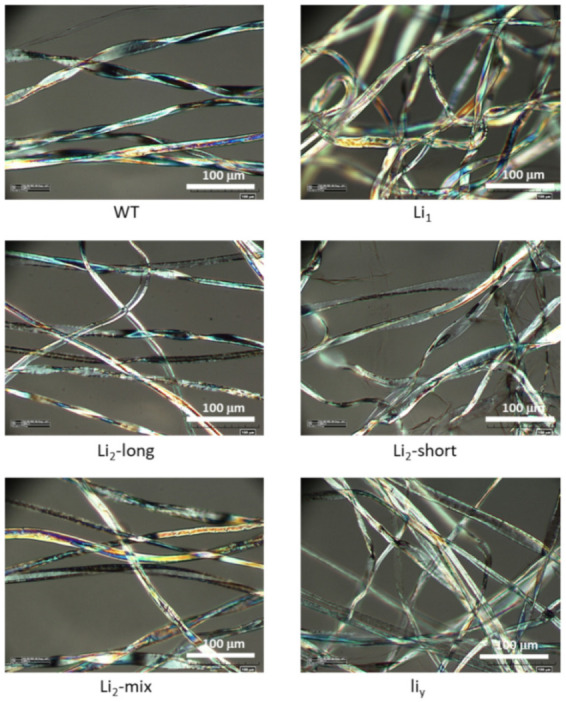
Morphologies of cotton fibers visualized by polarized optical microscopy.

The optical micromorphologic images are presented in Figure 3. The polarized optical microscopic analysis could be used to qualitatively evaluate the convolutions and birefringence intensity of the cotton fibers. The vibrant color contrast generated along the fiber represents the birefringence that was caused by the crystalline arrays of cellulose microfibrils in the secondary cell wall [23]. Thus, fibers with a well-organized secondary cell wall appeared brighter and show greater birefringence [24]. Less mature fibers appeared in blue color, whereas more mature fibers appeared in yellow color that represents a birefringence generated from organized crystalline cellulose [25]. According to the images of cotton fibers in Figure 1 and Figure 2, the birefringence intensity and fiber convolution were roughly in the order of WT ≥ *Li*_2_-*long* ≈ *Li*_2_-*mix* > *Li_1_* > *Li*_2_ *short* ≈ *liy*. The observed differences may reflect the hardship of these cotton fibers entering the secondary cell wall synthesis phase [26]. Thus, the morphological results were further tested by multiple spectroscopic analysis in as described in the sections below.

### 3.2. SEM and SEM–EDS Morphological Characteristics

**Figure 4:**
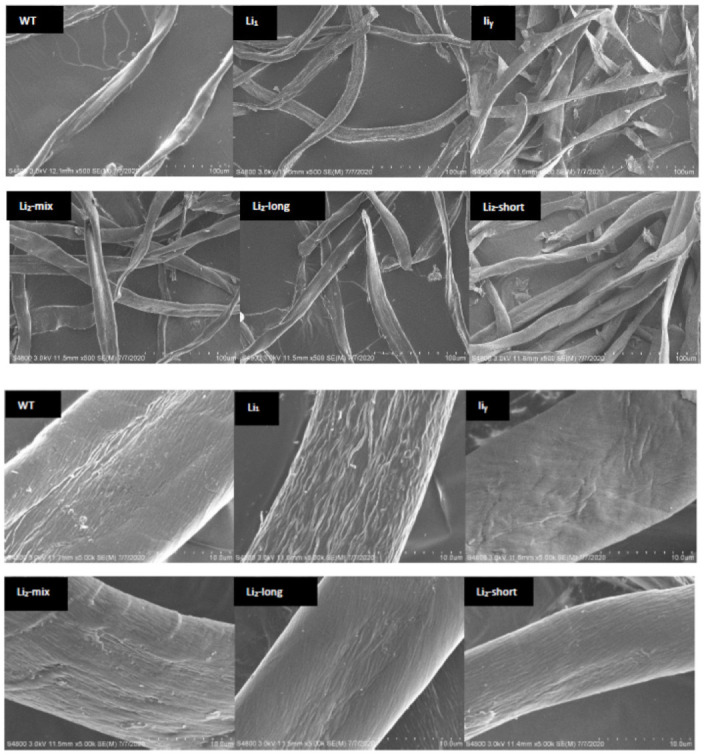
SEM images of six cotton fiber samples. The bar distance is 100 and 10 μm, respectively, for **upper** and **lower** panels.

Figure 4 shows the SEM images of the six cotton fibers in two scales. The morphology revealed by SEM imaging was typical for cotton fiber and cellulose samples [27,28]. In the meantime, some differences in the fiber shape and size distribution were observed among the six fibers we examined. The WT fibers were smoother, and not so warped (cursive) in the lateral direction as others. Compared to WT, the images of the other five samples suggested that there was some disruption of the out layer of the fibers. The surface of *Li*_1_ was full of trenches, and the surface of *li_y_* fibers was quite rough and cracking. Among the three *Li*_2_ samples, the surface of *Li*_2_-short fibers was close to that of *li_y_*. While the morphological features of *Li*_2_-mix and *Li*_2_-long looked more like those of WT fibers, there was still some disruption of the outer layer of the two *Li*_2_ samples.

**Figure 5:**
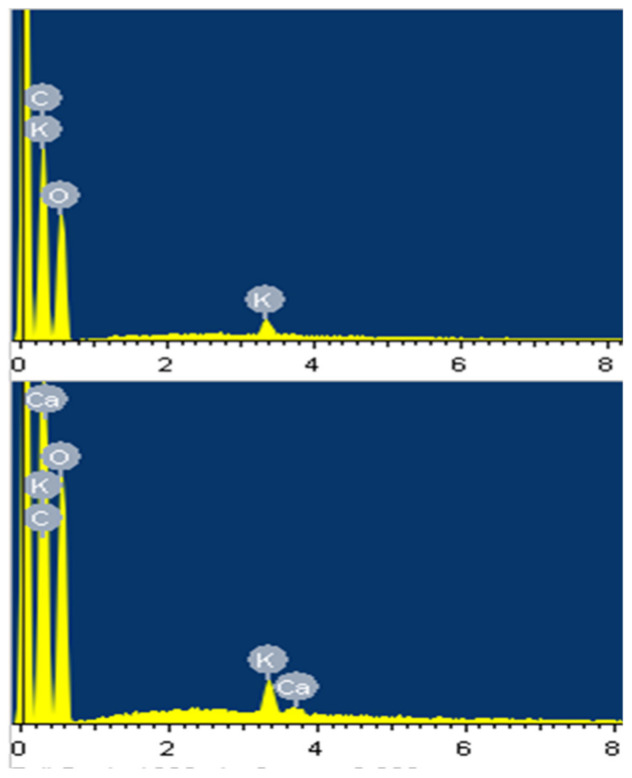
EDS spectrograms of WT (**upper**) and *li_y_* (**lower**) fiber samples.

Thus, the SEM–EDS were applied to detect the surface composition of the six samples. The representative spectrograms of WT and *li_y_* samples are presented in Figure 5. Strong peaks of C and O were detected in both samples as they should be the major elements of cotton fibers. Nitrogen, the representative protein component, was not able to be detected by EDS [29]. While the minor peaks of K appeared in both EDS spectrograms, the Ca peaks were detected only in *li_y_* spectrogram. Quantitative analysis showed that the content of the surface elements of *li_y_* and *Li*_2_-short fibers were similar (Table 1). The surface composition of *Li*_1_, *Li*_2_-mix, and *Li*_2_-long was more similar to WT with no Ca detected. Both K and Ca are essential plant nutrients [30,31]. They play important roles in cotton fiber elongation and maturity [32,33]. Guo et al. [33] studied the interaction of the two elements in modulating cotton fiber elongation. They analyzed the element contents of acid digests of the ovule and fiber samples collected at different developing stages by inductively coupled plasma–mass spectrometry. They found that a high accumulation of macroelements, but not Ca, was associated with longer fibers. Furthermore, using an in vitro ovule culture system, they demonstrated that Ca deficiency with appropriate K content, promoted cotton fiber elongation. Their transgenic cotton study suggested that a kinase gene-mediated the uptake of K under Ca-deficient conditions for fiber elongation. Our observation on the accumulation of Ca only on *li_y_* and *Li*_2_-shout mutant fibers was consistent with Guo et al. [33], and provided new clues on the genomic level to establishes links between Ca, K, and fiber elongation in these mutants. As those cottons were grown under the same fertilizer and management conditions, the overexpression of Ca-activity or regulatory genes may be one of the inhibitory factors of fiber elongation although vigorous research is needed to elucidate the relevant mechanisms. One hypothesis could be that Ca-enhanced rigid cell wall structures limited the fiber elongation as Ca can be a cell wall structural component and/or an environmental signaling molecule [33].

### 3.3. Wetting Behavior

**Figure 6:**
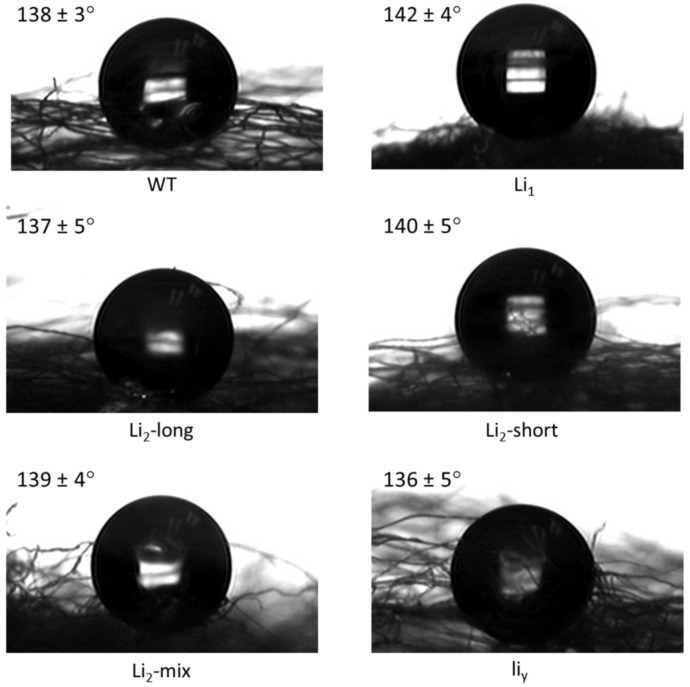
Water droplets on the fiber webs. The values at each image are the contact angle with standard deviation (*n* = 5). No statistically significant difference (*p* > 0.05) was observed between these values.

The wettability of the fiber samples was evaluated via water contact angle measurement. Quantitatively, the contact angles of the six samples were around 140° (Figure 6), similar to that in the literature [34]. These values were far greater than 90° which is considered to be hydrophobic, mainly due to the presence of waxes and pectin on the fiber surface [34]. Indeed, these fiber samples may be considered superhydrophobic with such high water contact angles [35]. However, the contact angle values were not significantly different (*p* > 0.05) between the six cotton fibers as their mean value is −139 ± 4 degrees.

### 3.4. FT-IR Analysis

**Figure 7:**
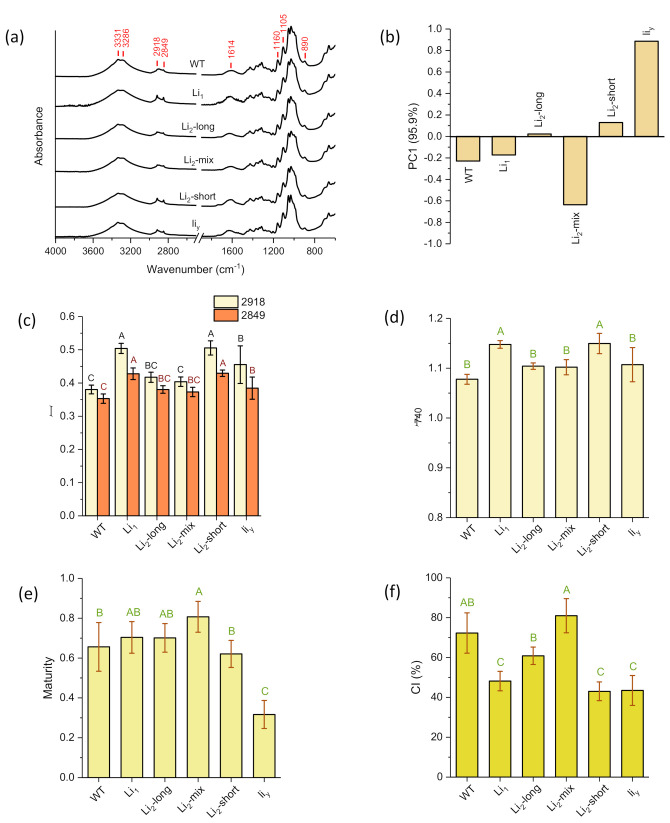
Attenuated total reflectance Fourier transform infrared (ATR FT-IR) spectra and relevant IR parameters of six cotton fiber samples. (**a**) Spectra; (**b**) the first principal component (PC1) scores of the six samples by PCA calculation of the normalized spectra; (**c**) the relative amounts of waxes semi-quantitatively measured by the normalized intensities of methylene peaks at 2918 and 2849 cm^−1^; (**d**) the relative amount of amorphous cellulose linked to the normalized intensity at 740 cm^−1^; (**e**) the IR-based maturity; and (**f**) IR-based cellulose crystallinity index (CI) calculated by three-band ratio algorithms. Data in the c–f are presented in average with SD bars (*n* = 5). Different letters indicate the values of the same parameter significantly different at *p* ≤ 0.05.

The ATR FT-IR spectral features of the six samples (Figure 7a) are typical for cotton fiber and other lignocellulosic biomass samples, and the general band assignments of the cotton fiber or cellulose are available in the literature [25,36,37]. These common features include a wide OH stretching band (3600–3000 cm^−1^), multiple CH stretching bands or shoulders (3000–2900 cm^−1^), the OH bending region (1800–1300 cm^−1^), and the fingerprint region with multiple combination bands (1250–850 cm^−1^) [38,39]. While the spectral features in the lower wavenumber range have been well studied [40,41], it is worthwhile discussing the bands around 3000–2800 cm^−1^ at the high wavenumber range. In agricultural chemistry research, the band intensity around this region is frequently evaluated, qualitatively or quantitatively, as a measure of the wettability or hydrophobicity of the testing samples [42,43,44] as these bands are contributed by aliphatic C–H stretching of fats and lipids [45]. These bands are also observed in cotton fibers arising from the C–H group of both cellulose and waxes [36,46]. Indeed, a pioneering effort calculated the intensity ratio of the 1372 cm^−1^ band against 2900 cm^−1^ to estimate the cellulose crystallinity from the IR measurement [47]. While the pioneering approach is not widely adopted, a recent work [48] examined in detail the band intensities of the developing cotton fibers in the high wavenumber region of 2800–3000 cm^−1^ using micro-FT-IR. The recent research assigned more specifically the absorbance at 2920 and 2850 cm^−1^ to CH_2_ asymmetrical and symmetrical stretching of waxes, respectively, and that at 2900 cm^−1^ to CH stretching of cellulose. In this way combined with the curve fitting method, they calculated the IR absorbance ratios of 2900 cm^−1^/2850 cm^−1^ and 2900 cm^−1^/2920 cm^−1^. While the two ratios are alternative measures of crystalline cellulose, the authors named them as the wax crystallinity index (WCI) due to the use of wax-relevant peaks as references. Thus, the two peaks of 2942 and 2874 cm^−1^ in Figure 7a indicated the presence of waxes on the six cotton fiber samples.

For an understanding of the similarity or dissimilarity of spectral features of the six fiber samples, these spectra were subjected to PCA recognition in the whole spectral region from 4000–600 cm^−1^. The analysis showed the dominated first principal component (PC1), accounting for 95.9% of the total variation (Figure 7b). The PC1 score increased from −0.64 to 0.89 in the order of *Li*_2_-mix < WT < *Li*_1_ < *Li*_2_-long < *Li*_2_-short < li_y_. Liu and Kim [2] reported that their PC1 scores increased with the increasing cellulose content in the developing cotton fiber. However, they conducted the PCA analysis which only used the ATR FT-IR spectra at the lower wavenumber ranged from 1800 to 600 cm^−1^. With the whole spectral PCA analysis, cellulose and other high wavenumber components (e.g., pectin and wax) could have contributed to PC1 scores [49,50]. Per PC1 scores, the six fiber samples could be separated into a negative score group (*Li*_2_-mix, WT, and *Li*_1_) and a positive score group (*Li*_2_-long, *Li*_2_-short, and *li_y_*). This grouping is similar to the morphologic observations of SEM and SEM–EDS. Thus, in addition to cellulose content, the changing trend and grouping of the PC1 scores of the six samples should have been evidence of the different development of fiber cell walls including both thickening and maturation [51].

Furthermore, four specific IR-based fiber parameters were calculated per the relevant individual peak intensities (Figure 7c–f). The changing trends of the band intensity at 2918 and 2849 cm^−1^ (for wax), and 740 cm^−1^ (for amorphous cellulose) are similar. The highest absorbance was observed with *Li*_1_ and *Li*_2_-short samples. The lowest intensity was with wild-type (WT) samples, but no or barely any significant difference with *Li*_2_-long, *Li*_2_-mix, and *li_y_* at *p* = 0.05. This observation may suggest that wax and amorphous cellulose are co-present in certain structural formats in the fiber synthesis and elongation processes. The highest values of both IR-based maturity and CI were observed with *Li*_2_-mix, and the lowest values with *li_y_*. However, no similarity was observed with the other four samples with regard to the two parameters. This result implied that the genetic mutation did not impact IR-based maturity and CI (i.e., the fiber growth) in the same way/mechanism among these mutant cottons as the two factors are reported as good parameters in monitoring fiber maturing processes [2,40].

### 3.5. Thermogravimetric Analysis

**Figure 8:**
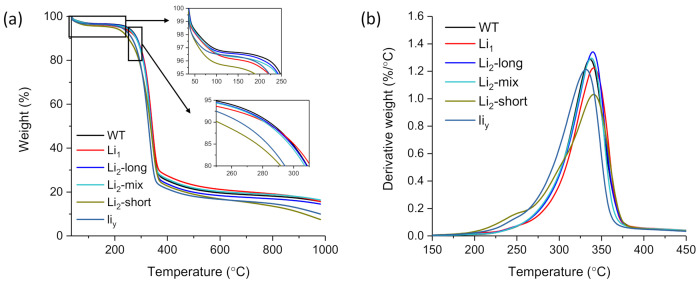
Thermograms of thermogravic analysis: (**a**) TG plots; (**b**) DTG plots.

The TG and DTG plots of the six fiber samples are presented in Figure 8. The TG curves show the typical three-stage weight loss of fiber samples [34]. These fibers first lost moisture at around 100 °C in the first stage. Then, the decomposition of cellulose occurred at 320–380 °C and decomposition of char above 380 °C. At higher temperatures, a rapid volatilization accompanied by the formation of levoglucosan which breaks down into smaller molecules (e.g., furans, aldehydes, ketones, aromatic hydrocarbons). Further repolymerization of these volatiles leads to the formation of char [52]. The initial weight loss at 250–300 °C might have also involved the degradation of hemicelluloses and other noncellulose components [49,53]. Such hemicellulose shoulder in the DTG curves was observed in *Li*_2_-short and *li_y_* samples. It was not obvious in the DTG curves of the other four samples. In other words, there were trace or small amounts of those noncellulose components in the matured WT, *Li*_1_, *Li*_2_-long, and *Li*_2_-mix fibers. Recently, Mironova et al. [54] investigated the structure and thermal behavior of cellulose and composite precursors with additives of silyl-substituted acetylene and alkoxysilanes. They examined and compared the TG curve shapes of their samples with great details in the lower pyrolysis temperature regions from 50 to 250 °C and from 265–310 °C. Their observations indicated that 5% of the additive is sufficient to greatly impact the carbon yield and other parameters compared to the non-additive cellulose sample. While there was no external additives in our samples, part of the differences in Figure 8 might be attributed to the internal noncellulose components (e.g., mineral Ca, K, and organic impurity) in addition to the effect of different crystability degrees in the six samples.

From the thermogravimetric analysis, eight quantitative parameters were determined (Table 2). The first two parameters were less impacted by the genetic mutation as their values were not so statistically significantly (*p* ≤ 0.05) diverse than other parameters. Compared to the data of the wild type (WT), the values of the other six parameters of the five mutant fibers either increased or decreased but not in consistent ways, indicating the different mutating mechanisms of these samples. One pair of six values of char yield (*Li*_2_-short and *li_y_*) were statistically significantly the same (*p* > 0.05), and other char yields significantly different from each other (*p* ≤ 0.05). The difference might not only be due to the char from the decomposition of the “dehydrocellulose” [39], but also due to non-volatile mineral residues with some shown in Table 1.

### 3.6. Correlation Analysis of the Two Sets of ATR FT-IR and Thermogravimetric Measurements

Correlation analysis has been used to gain more insight into the relationships between different tested parameters in agricultural studies [30,31,55]. Through correlation coefficient analysis, Waldrip et al. [56] reported that the FT-IR intensities at 2917, 2846, and 1650 cm^−1^ were positively correlated (*p* ≤ 0.05) to certain C and N forms in cattle manure samples. A recent work [57] reported that an FT-IR three-band (1800, 1700, and 650 cm^−1^) R reading, developed for agricultural biomass-based biochars [58], were linearly related to the pyrolysis temperature in preparation of cottonseed meal-based biochars. Thus, in this work, we calculated the correlation coefficients between the two sets of data obtained from ATR FTIR and thermogravimetric analysis (Table 3). There were five pairs of data between the two measurements showing the correlation coefficients significant at *p* ≤ 0.05. The PC1 from the PCA analysis of FT-IR data was negatively correlated to the weight loss at onset decomposition (*WL_o_*). Four significant coefficients were related to the FT-IR-based maturity parameter. On the thermal properties side, the onset decomposition temperature and all three weight loss parameters (i.e., *WL_o_*, *WL_p_*, *WL_e_*) were correlated to the FT-IR maturity degrees although the onset decomposition temperature and weight loss were negatively correlated to the maturity degrees. This observation suggested that the maturity degree was strongly linked to the thermal stability (or decomposition/degradation potential). While there were only six samples for the analysis, there were also a few other coefficient values close to the critical 0.811 (significant at *p* = 0.05). In the current analysis, there was no significant (*p* > 0.05) correlation observed between the IR-based CI and thermal properties. Even though there are reports on the high consistence between IR-based CI and X-ray diffraction-measured CI [21,48,59], it may be worthwhile, in future research, exploring whether there is any possible correlations of these thermal behaviors with X-ray diffraction parameters of crystallinity and crystal size [51].

## 4. Conclusions

This work comparatively investigated the surface and thermal characteristics of six cotton fiber samples collected from a wild-type (WT) and three lintless mutants (i.e., *Li*_1_, *Li*_2_, and *li_y_*). Apart from the water contact angles (wetting behaviors), the impacts of genetic mutation on all other measured parameters were observed in the five mutant samples. The fiber micromorphology, surface composition, and thermal stability of *Li*_2_-long and *Li*_2_-mix were somehow closer to those of WT. On the other hand, *Li*_2_-short and *li_y_* were more similar to each other, far different from those of WT, while *Li*_1_ was in the middle of the spectrum. Among the five FT-IR based parameters, only the maturity parameter was well correlated (*p* ≤ 0.05) to the onset decomposition temperature and all three weight-loss parameters at onset, peak, and end decomposition stages out of the eight thermal properties measured. This observation suggested the IR-based maturity was a better parameter than CI and other FT-IR parameters that reflect the thermal stability (i.e., decomposition/degradation potential) of cotton fiber. The distinct presence of Ca in the surface composition of naturally occurred mutant *Li*_2_-short and human-made mutant *li_y_* suggested that Ca-enhanced rigid cell wall structures limited the fiber elongation in the two mutants as Ca can be a cell wall structural component and/or an environmental signaling molecule. In other words, the overexpression of Ca-activity or regulatory genes may be one of the inhibitory mechanisms of fiber elongation. While the elongation inhibitory mechanisms should be different in another three naturally occurring mutants, no clear evidence was found to link their phenotype properties measured in this study to any potential genetic mutation clues.

## Figures and Tables

**Table 1:** Elemental composition in weight (*W*%) and atomic (*A*%) percentages of the cotton fiber samples by SEM–EDS analysis. Number of iterations = 5. Weight % difference (systematic error) < 0.1%.

Element	WT		*Li*_1_		*li*_y_		*Li*_2_-*long*		*Li*_2_-*medium*		*Li*_2_-*short*	
	*W*	*A*	*W*	*A*	*W*	*A*	*W*	*A*	*W*	*A*	*W*	*A*
C	51.45	58.67	52.49	59.63	53.34	60.54	49.94	57.20	51.34	58.53	52.39	60.15
O	48.08	41.16	47.20	40.26	46.09	39.27	49.57	42.62	48.30	41.34	46.47	39.65
K	0.47	0.16	0.31	0.11	0.47	0.17	0.50	0.18	0.36	0.12	0.44	0.15
Ca	ND ^1^	ND	ND	ND	0.10	0.03	ND	ND	ND	ND	0.16	0.05

^1^ not detected.

**Table 2:** Comparison of thermogravimetric data from the TG and DTG analysis.

	*T_o_* (°C)	*WL_o_* (%)	*T_p_* (°C)	*WL_p_* (%)	*D*_p_(%/°C)	*T_e_* (°C)	*WL_e_* (%)	Char (%) ^C^
WT	251.4 ^A^ (0.1) ^B^	5.8 ^A^ (0.5)	335.9 *D* (0.4)	47.5 ^C^ (1.0)	1.256 ^BC^ (0.031)	360.4 ^C^ (0.1)	69.4 ^C^ (0.5)	19.6 ^C^ (0.5)
*Li*_1_	248.5 ^A^ (3.8)	6.1 ^A^ (0.4)	341.2 ^A^ (0.4)	47.5 ^C^ (0.8)	1.217 ^C^ (0.007)	363.1 ^B^ (0.785)	68.1 ^D^ (0.6)	21.1 ^A^ (0.1)
*Li*_2_-*long*	249.1 ^A^ (0.6)	5.6 ^A^ (0.1)	339.6 ^B^ (0.1)	51.3 ^B^ (0.5)	1.328 ^A^ (0.014)	360.3 ^C^ (0.2)	71.4 ^B^ (0.1)	18.3 *D* (0.3)
*Li*_2_-*mix*	252.3 ^A^ (1.3)	5.8 ^A^ (0.1)	338.2 ^C^ (0.2)	49.8 ^B^ (0.4)	1.288 ^AB^ (0.010)	358.6 *D* (0.4)	69.3 ^C^ (0.3)	20.4 ^B^ (0.1)
*Li*_2_-*short*	209.7 ^B^ (0.2)	5.4 ^A^ (0.3)	340.9 ^A^ (0.7)	54.7 ^A^ (0.6)	1.066 *D* (0.035)	366.6 ^A^ (0.9)	74.2 ^A^ (0.4)	16.8 *_e_* (0.1)
*li_y_*	210.5 ^B^ (0.5)	4.5 ^B^ (0.1)	331.4 *_e_* (0.4)	54.3 ^A^ (0.1)	1.206 ^C^ (0.008)	353.8 *_e_* (0.1)	74.2 ^A^ (0.2)	16.7 *_e_* (0.1)

*T*: temperature; *WL*: weight loss; *D*: decomposition rate; *_o_*: onset decomposition; *_p_*: peak decomposition; *_e_*: end decomposition. ^A^: averages followed by different letters are significantly different (*p* < 0.05); ^B^: standard deviation of three measurements; ^C^: char yield measured at 600 °C.

**Table 3:** Correlation coefficients between the ATR FT-IR-based parameters and thermal properties of the six fiber samples.

	*PC1*	*I*_2918_	*I*_2849_	*I*_740_	*Maturity*	*CI*
*T*_0_	−0.790	−0.571	0.664	−0.415	−0.942 **	0.767
*WL*_0_	−0.889 *	−0.087	0.761	0.104	−0.875 *	0.485
*T_p_*	−0.596	0.386	0.631	0.601	−0.478	0.001
*WL_p_*	0.690	0.354	−0.689	0.286	0.886 *	−0.575
*D_p_*	−0.352	−0.766	0.354	−0.680	−0.620	0.680
*T_e_*	0.178	0.586	0.236	0.697	0.410	−0.452
*WLe*	0.780	0.303	−0.782	0.182	0.866 *	−0.606
*Char*	−0.791	−0.219	0.804	−0.084	−0.793	0.573

Symbol *, ** indicates the coefficient value significant at *p* = 0.05, and 0.01, respectively.

## Data Availability

Not applicable.
